# Antidepressant use in relation to dementia risk, cognitive decline, and brain atrophy

**DOI:** 10.1002/alz.13807

**Published:** 2024-04-01

**Authors:** Ilse vom Hofe, Bruno H. Stricker, Meike W. Vernooij, M. Kamran Ikram, M. Arfan Ikram, Frank J. Wolters

**Affiliations:** ^1^ Department of Epidemiology Erasmus University Medical Center Rotterdam The Netherlands; ^2^ Department of Radiology & Nuclear Medicine and Alzheimer Centre Erasmus MC Erasmus University Medical Center Rotterdam The Netherlands; ^3^ Department of Neurology Erasmus University Medical Center Rotterdam The Netherlands

**Keywords:** antidepressant use, cognitive decline, depression, MRI, population‐based

## Abstract

**INTRODUCTION:**

We aimed to assess the effect of antidepressant use on dementia risk, cognitive decline, and brain atrophy.

**METHODS:**

In this prospective cohort study, we included 5511 dementia‐free participants (Mini‐Mental State Examination [MMSE] > 25) of the Rotterdam study (57.5% women, mean age 70.6 years). Antidepressant use was extracted from pharmacy records from 1991 until baseline (2002–2008). Incident dementia was monitored from baseline until 2018, with repeated cognitive assessment and magnetic resonance imaging (MRI) every 4 years.

**RESULTS:**

Compared to never use, any antidepressant use was not associated with dementia risk (hazard ratio [HR] 1.14, 95% confidence interval [CI] 0.92–1.41), or with accelerated cognitive decline or atrophy of white and gray matter. Compared to never use, dementia risk was somewhat higher with tricyclic antidepressants (HR 1.36, 95% CI 1.01–1.83) than with selective serotonin reuptake inhibitors (HR 1.12, 95% CI 0.81–1.54), but without dose–response relationships, accelerated cognitive decline, or atrophy in either group.

**DISCUSSION:**

Antidepressant medication in adults without indication of cognitive impairment was not consistently associated with long‐term adverse cognitive effects.

**Highlights:**

- Antidepressant medications are frequently prescribed, especially among older adults.
- In this study, antidepressant use was not associated with long‐term dementia risk.
- Antidepressant use was not associated with cognitive decline or brain atrophy.
- Our results support safe prescription in an older, cognitively healthy population.

## 1 INTRODUCTION

Dementia is characterized by impairment in multiple cognitive domains, causing large burden on patients,^1^ caregivers, and society.^2^ Use of antidepressant medication has been suggested to contribute to dementia pathology through adverse anticholinergic and vascular effects, thereby accelerating cognitive decline and increasing incidence of dementia (dementia risk).^3^, ^4^ Older adults may be more susceptible to these adverse effects due to age‐related alterations in pharmacokinetics and pharmacodynamics, including reduced acetylcholine‐mediated transmission in the brain and increased blood–brain barrier permeability.^5^ Approximately 10% of the population in high‐income countries use antidepressants, with more frequent use at older ages,^6^, ^7^ and this percentage has more than doubled over past decades.^6^ Despite the frequent prescription of antidepressants, their long‐term effects remain uncertain, as short‐term follow‐up in clinical trials generally precludes the investigation of chronic use and long‐term adverse outcomes.^8^ Long‐term observational data are needed, but cohort studies to date have produced contrasting results. While various studies observed increased risk of dementia in antidepressant users,^4^, ^8^, ^9^, ^10^, ^11^, ^12^, ^13^, ^14^ several others did not.^15^, ^16^, ^17^, ^18^, ^19^

These studies have been challenged methodologically by the close relation between depression and dementia. Depression is a well‐established risk factor of dementia,^20^ and the prescription of antidepressant medication in patients with depressive symptoms might link antidepressant use to dementia even in the absence of a true causal association (i.e., confounding‐by‐indication). In addition, depressive symptoms often occur in the prodromal phase of dementia, as a psychological reaction to cognitive impairment or due to shared neurobiological mechanisms between neurodegenerative diseases and depression.^21^ In this case, prodromal symptoms of neurodegeneration give rise to antidepressant use, which is commonly referred to as reversed causality. The concepts of confounding‐by‐indication and reversed causality make it difficult to distinguish between truly causal effects of antidepressants on neurodegeneration, and increased antidepressant prescription in response to a shared risk factor or prodromal neurodegenerative disease. This question of causality could be further investigated by determining drug effects on preclinical markers of neurodegeneration or by using an instrumental variable approach (e.g., markers of drug metabolism), but few published studies have examined the effect of antidepressant use on cognitive decline,^11^, ^16^ and to our knowledge only one study examined the long‐term effects of antidepressant use on brain atrophy.^22^

We, therefore, aimed to estimate the effect of antidepressant use on dementia risk, cognitive decline, and brain atrophy in a longstanding population‐based cohort study.

**RESEARCH IN CONTEXT**

1. **Systematic review**: Clinical trials on antidepressant use and possible adverse cognitive effects are often limited in their follow‐up duration, which makes it challenging to assess the effect of chronic use and long‐term outcomes of antidepressant use. Previous long‐term observational studies investigating the possible lasting effects of antidepressant use on cognition produced conflicting results, and there is a scarcity of data on the effect on magnetic resonance imaging (MRI) ‐markers of neurodegeneration.
2. **Interpretation**: Our findings showed that in a cognitively healthy population, antidepressant use was not consistently associated with long‐term dementia risk, accelerated cognitive decline, or brain atrophy of the gray matter, white matter, hippocampus, thalamus, or amygdala. This effect was independent of dose or duration of use.
3. **Future directions**: Future research should focus on possible lasting effects of antidepressant use in older adults who are more vulnerable to adverse effects, for example, in a memory clinic population.

## 2 METHODS

### 2.1 Study population

This study was embedded in the Rotterdam Study, an ongoing prospective population‐based cohort study, the details of which have been described previously.^23^ Briefly, the Rotterdam Study started in 1990 with 7983 participants 55 years of age and older. The original cohort (RS‐I) was extended in 2000 with 3011 participants (RS‐II). In 2006, an additional 3932 participants 45 years of age and older were added as a third recruitment wave (RS‐III). This resulted in the inclusion of 14,926 participants, who undergo follow‐up examinations at a dedicated research center every 4 years. For the incident dementia and cognition analyses, we included participants who took part in the fourth visit of RS‐I (2002–2004), the second visit of RS‐II (2004–2005), or the first visit of RS‐III (2006–2008), who were at risk of dementia and 60 years of age or older during this visit. Of 6953 eligible participants, we excluded those with an indication of cognitive impairment at baseline (*N* = 1.442). Cognitive impairment was defined as a score of < 26 on the Mini–Mental State Examination (MMSE).^24^ This resulted in the inclusion of 5511 participants. An overview of the inclusion of participants is presented in Figure S1. For the magnetic resonance imaging (MRI) analyses similar inclusion criteria were applied, with the exception of age restriction. Given the long preclinical phase of neurodegenerative disease, all participants >45 years were allowed to partake in this analyses, which resulted in 8017 eligible participants, of whom 5303 (66.1%) underwent brain MRI and 4912 had at least one scan that passed quality control.

### 2.2 Use of antidepressant medication

Pharmacy records were available from 1991 onward for cohort RS‐I, and from 1995 onward for cohort RS‐II and cohort RS‐III. Pharmacy records provided detailed information on prescription date and defined daily dose on a day‐to‐day basis, classified according to the Anatomical Therapeutic Chemical (ATC) code. To limit reversed causation (i.e., the use of antidepressants for depression due to prodromal dementia), antidepressant use was defined as prescription of any antidepressant during the exposure period, which is between inception of pharmacy records (1991) and study baseline (2002–2008). An overview of the study design is represented in Figure S1. Registry data were complete for all participants.

### 2.3 Dementia screening and surveillance

Participants were screened for dementia at each center visit, using the Mini‐Mental State Examination (MMSE) and the Geriatric Mental Schedule (GMS). Participants with MMSE <26 or GMS >0 underwent further investigation, including an informant interview and the Cambridge Examination for Mental Disorders of the Elderly (CAMDEX).^25^ In addition, the entire cohort was continuously under surveillance for dementia through electronic linkage with medical records from general practitioners and the regional institute for outpatient mental health care. All cases that were suspicious for dementia were reviewed by a consensus panel, including a consultant neurologist, which applied standard criteria for dementia (Diagnostic and Statistical Manual of Mental Disorders, Fourth Edition [DSM‐IV]) to come to a final diagnosis. Follow‐up for dementia until January 1, 2018, was complete for 96.8% of the potential person‐years. Participants were censored at date of dementia diagnosis, date of death, date of loss to follow‐up, or January 1, 2018, whichever came first.^25^

### 2.4 Cognitive assessment

Cognitive functioning was assessed at baseline and follow‐up visits with a neuropsychological test battery comprising the letter‐digit substitution task (LDST; number of correct digits in 1 minute),^26^ the verbal fluency test (animal categories),^27^ the Stroop test (error‐adjusted time in seconds),^28^ the 15 word learning test (immediate and delayed recall),^29^ and the Purdue Pegboard Test.^30^ To obtain a measure of global cognitive functioning, a standardized compound score (g‐factor) was calculated using the first factor of a principal component analysis, including each of the aforementioned cognitive tests, which explained 49.5% of the variance in cognitive scores. In the event that data on the individual cognitive tests were missing, these data were imputed using single imputation, based on age, sex, education, MMSE score, and other available cognitive tests.

### 2.5 MRI protocol and image processing

Throughout the study period, MRI of the brain was performed on the same 1.5T scanner (General Electric Healthcare, Milwaukee, WI) using an 8‐channel head coil. Imaging acquisition included a high‐resolution, three‐dimensional (3D) T1‐weighted, proton density weighted and a fluid‐attenuated inversion recovery (FLAIR) sequence. A detailed protocol of the Rotterdam Scan Study is described elsewhere.^31^ Volumes in milliliters (mL) of the total brain, gray matter, and white matter was obtained by automated tissue segmentation based on a k‐nearest neighbor algorithm. All segmentations were visually inspected and manually corrected when necessary. Volumes of the hippocampus, thalamus, and amygdala were obtained by processing T1‐weighted images with FreeSurfer (version 6.0).^32^

### 2.6 Genotyping

DNA genotyping was performed on collected blood samples and conducted with 550K, 550K duo, or 610K Illumina arrays at the Erasmus MC, Rotterdam. Single nucleotide polymorphisms (SNPs) were imputed to the Haplotype Reference Consortium using the Michigan Imputation Server (version 1.0) and the Minimac software.^33^ For the instrumental variable analyses, we focused on the two most used antidepressants, amitriptyline and paroxetine. Based on the established involvement of the cytochrome P450 (CYP)2D6 genotype in the metabolization of amitriptyline and paroxetine,^34^ we used a CYP2D6‐based unweighted genetic risk score (GRS) as instrumental variable. In the construction of the GRS we included all available SNPs with a prevalence >1% in participants of European ancestry, or >10% in those of African or Asian ancestry (because of the small number of non‐White participants in the Rotterdam Study). Five SNPs were missing (rs35742686, rs1065858, rs5030655, rs5030656, and rs28371716), one of which (rs5030655) could be replaced with a proxy (rs139779104, r^2^ ≥0.8); the other four were excluded from the analysis. We excluded one additional SNP because of high linkage disequilibrium with the other variants (rs1065852). The remaining 23 SNPs were translated to star alleles using the PharmVar translation table.^35^ The GRS comprised the sum of all present SNPs.

### 2.7 Other measurements

Information on age, sex, educational attainment (primary, lower, intermediate, or higher education), smoking habits (never, current, or former), and alcohol use (no use, ever use) was ascertained at baseline during home interview. Prevalence of stroke, parkinsonism, cancer, coronary heart disease, congestive heart failure, atrial fibrillation, and chronic obstructive pulmonary disease was assessed by interview at baseline and verified in the medical records. The Center for Epidemiology Depression Scale (CES‐D) was used to assess depressive symptoms at baseline.^36^ Height and weight were measured from which the body mass index [BMI] kg/m^2^) was computed. Blood pressure was measured in sitting position using a random‐zero sphygmomanometer. Hypertension was defined as a systolic blood pressure >140 mmHg, a diastolic blood pressure >90 mmHg or use of antihypertensive medication. The estimated glomerular filtration rate (eGFR) was calculated using the Chronic Kidney Disease Epidemiology Collaboration equation, based on creatinine concentrations in fasting blood samples.^37^ Diabetes was defined as fasting blood glucose >7.0 mmol/L or use of antidiabetic medication. Use of benzodiazepines and antipsychotic medication was extracted from pharmacy records.

### 2.8 Statistical analyses

Missing covariate data (maximum 10%) was imputed using 10‐fold multiple imputation; all covariates were included as predictors in the model. Distribution of variables was similar in the imputed and non‐imputed datasets. For the main analyses, we compared ever‐use of antidepressant medication to never use. Further analyses were performed distinguishing former from current use and defining exposure as duration of use and cumulative defined daily dose. Due to the right skewed distribution of these variables, we categorized the variables (duration of use: none vs short‐term [1–90 days] vs long‐term [>90 days], and cumulative defined daily dose: none vs = < median vs > median).

First, we estimated the association between antidepressant use and risk of all‐cause dementia using Cox proportional hazards regression models. All analyses were adjusted for age, sex, and education (Model 1), and additionally for smoking habits, alcohol use, BMI, eGFR, CES‐D score, benzodiazepine use, antipsychotic use, and prevalence of diabetes, hypertension, stroke, parkinsonism, atrial fibrillation, congestive heart failure, coronary heart disease, cancer, and chronic obstructive pulmonary disease (Model 2). We repeated the analyses for clinical Alzheimer's Disease (AD) only, and separately for tricyclic antidepressants (TCAs) and serotonin reuptake inhibitors (SSRIs) as the most used types of antidepressants. To assess confounding by indication, we further stratified analyses by history of depression and severity of depressive symptoms according to CES‐D score (cutoff: ≥16).

We then estimated differences in cognition between antidepressant users and non‐users at baseline using linear regression models, and the association between antidepressant use and change in cognition in all participants with at least one cognitive test assessment (*N* = 5331/5511 [96.7%]), using linear mixed models. The linear mixed models included random slopes for the effect of follow‐up time, and a quadratic term for age. Adjustments were similar to the dementia models. We again repeated the analyses for TCAs and SSRIs, and for each cognitive test separately.

Next, we applied linear regression models to estimate baseline differences in brain volume between users and non‐users. The association of antidepressant use with change in brain volume over time of the total brain, white matter, gray matter, and subcortical structures involved in memory and mood regulation (i.e., hippocampus, amygdala, and thalamus ^38^) were estimated using linear mixed models, adding total intracranial volume to the aforementioned set of covariates. Analyses were again repeated separately for antidepressant type.

Finally, we performed an instrumental variable analysis to strengthen the causal inference of associations with dementia risk. Among users of amitriptyline and paroxetine, we estimated the effect of rate of drug metabolism (according to CYP2D6 genotype) on dementia risk. We used Cox regression models, adjusting for age and sex (Model 1), and additionally for median daily dose and use of beta‐blockers, benzodiazepines, antipsychotic medication, and alternative types of antidepressants (Model 2).

All analyses were done using SPSS version 28^39^ and R version 4.0.2 (packages: ‘Mice,’^40^ ‘nlme’^41^).

## 3 RESULTS

Baseline characteristics of the study population are presented in Table 1. Among 5511 participants, 923 (16.7%) had used antidepressants in the ≈10‐year period prior to baseline, of whom 227 (4.1%) were using at study baseline. At baseline, medication use was more frequent in women than in men (20.8% vs 11.7%) and in lower educated individuals, and it increased with age from 2.1% for those 45–50 years of age to 5.4% after age 80.

**TABLE 1:** Baseline characteristics of the study cohort.

Characteristics	Total study population(*N* = 5511)	MRI sample (*N* = 4912)
Age, years	70.6 (± 7.6)	63.5 (± 10.8)
Women	3169 (57.5%)	2736 (55.7%)
Education		
Primary	537 (9.7%)	382 (7.8%)
Lower/intermediate or lower vocational	2370 (43.0%)	1830 (37.3%)
Intermediate vocational or higher	1661 (30.1%)	1501 (30.6%)
Higher vocational or university	870 (15.8%)	1152 (23.5%)
Body mass index, kg/m^2^	27.7 (± 4.2)	27.4 (4.10)
Smoking		
Never	1581 (28.7%)	1464 (29.8%)
Former	2523 (45.8%)	1272 (25.9%)
Current	1308 (23.7%)	2110 (43.0%)
Alcohol	4694 (85.2%)	4300 (87.5%)
Diabetes	713 (12.9%)	616 (12.5%)
Hypertension	4205 (76.3%)	3047 (62.0%)
Stroke	127 (2.3%)	113 (2.3%)
Parkinsonism	20 (0.4%)	12 (0.2%)
Coronary heart disease	509 (9.2%)	308 (6.3%)
Heart failure	239 (4.3%)	65 (1.3%)
Atrial fibrillation	314 (5.7%)	136 (2.8%)
Cancer	519 (9.4%)	489 (10.0%)
Chronic obstructive pulmonary disease	434 (7.9%)	275 (5.6%)
Glomerular filtration rate	74.7 (14.5)	82.3 (15.8)
Center for Epidemiologic Studies Depression Scale ≥16	511 (9.3%)	401 (8.2%)
Benzodiazepine use	2737 (49.7%)	2354 (47.9%)
Antipsychotic medication use	87 (1.6%)	89 (1.8%)

*Note*: Data are presented as frequency (%) for categorical, and mean ± SD for continuous variables.

Covariates with missing data in total study population: education (1.3%), body mass index (5.1%), smoking (1.8%), alcohol (1.8%), diabetes (3.4%), hypertension (0.8%), coronary heart disease (1.7%), heart failure (0.2%), atrial fibrillation (8.5%), cancer (0.1%), chronic obstructive pulmonary disease (0.1%), glomerular filtration rate (5.8%), Center for Epidemiologic Studies depression Scale (2.7%).

Of all ever‐users, 309 (33%) had solely used TCAs, 335 (36%) had used SSRIs, 45 (5%) had used other antidepressants, and 233 (25%) had used a combination of aforementioned.

### 3.1 Incident dementia

**FIGURE 1:**
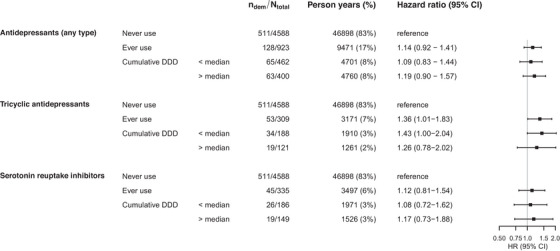
Antidepressant medication use and risk of all‐cause dementia. Estimates shown are from the fully adjusted Model 2, which is adjusted for age, sex, education, smoking status, alcohol use, body mass index, estimated glomerular filtration rate, Center for Epidemiologic Studies Depression scale score, benzodiazepine use, antipsychotic medication use, as well as prevalence of diabetes, hypertension, stroke, parkinsonism, atrial fibrillation, congestive heart failure, coronary heart disease, cancer, and chronic obstructive pulmonary disease. The median daily dose in tricyclic antidepressant users was 39.6 DDD, and in selective serotonin reuptake inhibitor users 315.0 DDD. N_dem _, number of dementia cases; N_total_, total number of participants in group; CI, confidence interval; DDD, defined daily dose.

During a mean follow‐up of 10.2 years, 639 individuals (11.6%) developed dementia. In age‐ and sex‐adjusted models, use of any antidepressant medication was associated with a mild to moderate increase in dementia risk (hazard ratio [HR] 1.24, 95% confidence interval [95% CI] 1.02–1.51), but risk estimates attenuated after adjustment for potential confounders. In the fully adjusted models, antidepressant use was not associated significantly with dementia risk (HR 1.14, 95% CI 0.92–1.41; Figure 1). There was no evidence of a dose–response relationship (Figure 1, Table S1).

With respect to antidepressant types, SSRI use was not associated with dementia risk (HR 1.12, 95% CI 0.81–1.54; Figure 1), irrespective of duration of use and cumulative dose (Figure 1, Table S1). Use of TCAs was associated with a mild to moderate increase in dementia risk (HR 1.36, 95% CI 1.01–1.83), but without evidence of a dose–response relationship (Figure 1, Table S1). Use of a combination of antidepressants was not associated with dementia risk (HR 0.91, 95% CI 0.60–1.38). The limited number of cases within this group prohibited stratification by dose or duration of use (Table S1).

Results were comparable for clinical AD only (HR 1.05, 95% CI 0.82–1.35). Effect estimates for all‐cause dementia were higher for current use than for past use (HR 1.38, 95% CI 0.99–1.93, for current use; and for past use: HR 1.05, 95% CI 0.82–1.34). Associations of antidepressant use with dementia risk were broadly similar between participants with or without (a history of) depression (HR 1.15, 95% CI 075–1.76, with depression; and without depression: 1.15, 0.90–1.47).

### 3.2 Cognition

**FIGURE 2:**
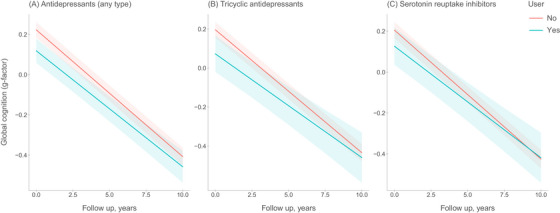
Antidepressant medication use and change in global cognition (g‐factor) over time. Trajectories show change in global cognition per year for ever users and never users. Global cognition is defined as the standardized compound score (g‐factor) using the first factor of a principal component analysis, including the letter‐digit substitution task, verbal fluency test, Stroop test, 15‐word learning test, and Purdue Pegboard Test. For graphical representation, trajectories are depicted for mean age, sex, education, and cohort; corresponding effect estimates in the main text refer to the fully adjusted models.

Of the 5331 participants who underwent cognitive assessment at baseline, 3517 (66.0%) had at least one repeated assessment at follow‐up (mean interval: 6.6 years). At baseline, global cognitive performance did not differ between antidepressant users and non‐users (g‐factor: β –0.03; 95% CI −0.09, 0.03). Antidepressant use was not associated with decline in global cognitive performance over time (mean difference in change in g‐factor per year: β 0.003; 95% CI −0.003, 0.010; Figure 2). Once again, we observed no dose–response relationship (Table S2). For the separate cognitive tests, we did not observe an effect of antidepressant use on long‐term performance on the letter‐digit substitution task, the Stroop test (time adjusted for errors on trial 3), the word fluency test, and the 15‐word learning test (delayed recall). Antidepressant use was associated with slowed reduction in performance on the Purdue Pegboard Test (mean difference in change in standardized cognitive test score per year: β [95% CI], for the letter‐digit substitution task: −0.002 [−0.008, 0.005], the Stroop test: −0.005 [−0.014, 0.004], the word fluency test: −0.003 [−0.012, 0.006], the word learning test: 0.003 [−0.006, 0.012], and the Purdue Pegboard Test: 0.010 [0.002–0.018]; Figure S2).

In participants who used only TCAs, no association was observed with cognitive decline (mean difference in g‐factor per year: β [95% CI]: 0.010 [−0.001, 0.021], Figure 2), irrespective of dose and duration of use (Table S2). Similar results were observed in participants who used only SSRIs (mean difference in g‐factor per year: β [95% CI]: 0.006 [−0.004, 0.017], Figure 2), irrespective of dose and duration (Table S2). Use of a combination of antidepressants was not associated with increased dementia risk, and no dose–response relationships were observed (Table S2).

### 3.3 Imaging‐derived brain volume

Of the 4912 participants who underwent MRI at baseline, 3588 (73.0%) had at least one repeated assessment (mean interval: 4.0 years). Participants without MRI were more often women, older, and less educated; had on average more depressive symptoms and a higher BMI; were more often diagnosed with hypertension, diabetes, stroke, coronary heart disease (CHD), congestive heart failure (CHF), atrial fibrillation (AF), cancer, and chronic obstructive pulmonary disease (COPD), were less often smokers and consuming alcohol, and had lower eGFRs than those with available MRI.

**FIGURE 3:**
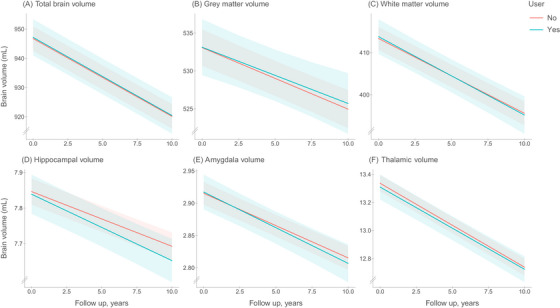
Antidepressant medication use and change in brain volumes in milliliters (mL) on MRI. Trajectories show change in mL brain volume per year for ever users and never users. For graphical representation, trajectories are depicted for mean age, sex, education, and cohort. Corresponding effect estimates in the main text refer to the fully adjusted models.

At baseline we observed no differences in brain volume between participants who had used antidepressant medication during the exposure period, and never users (β [95% CI]: total brain: 1.89 [−4.62, 8.39]; gray matter: 0.92 [−2.79, 4.62]; white matter: 0.97 [−3.24, 5.18]; hippocampus: 0.02 [−0.04, 0.07]; amygdala: 0.003 [−0.025, 0.031], thalamus: 0.01 [−0.08, 0.11]). Over time, no differences were observed between users and non‐users in change in brain volume in the total brain (β [95% CI]: 0.000 [−0.276, 0.276]), gray matter (0.066 [−0.312, 0.444]), white matter (−0.051 [−0.422, 0.321]), hippocampus (−0.004 [−0.008, 0.000]), amygdala (−0.001 [−0.004, 0.002]), or thalamus (0.002 [−0.005;0.0089]; Figure 3, Table S3). TCA and SSRI use were not associated with reduction in any of the areas of interest (Table 2).

**TABLE 2:** Antidepressant use (tricyclic antidepressants and selective serotonin reuptake inhibitors) and difference in change in mL brain volume over time.

Medication	Brain region	Model 1 Mean difference (95% CI)	Model 2 Mean difference (95% CI)
Tricyclic antidepressants (ever vs none)	Total brain	−0.101 (−0.507; 0.304)	−0.080 (−0.470; 0.310)
	Gray matter	−0.165 (−0.729; 0.398)	−0.076 (−0.604; 0.452)
	White matter	0.042 (−0.501; 0.585)	0.040 (−0.482; 0.562)
	Hippocampus	−0.000 (−0.006; 0.006)	−0.001 (−0.005; 0.007)
	Amygdala	0.001 (−0.003; 0.005)	0.001 (−0.003; 0.005)
	Thalamus	0.003 (−0.007; 0.012)	0.003 (−0.006; 0.013)
Selective serotonin reuptake inhibitors (ever vs none)	Total brain	−0.041 (−0.378; 0.296)	−0.018 (−0.345; 0.309)
	Gray matter	0.159 (−0.316; 0.633)	0.098 (−0.352; 0.549)
	White matter	−0.185 (−0.640; 0.270)	−0.133 (−0.574; 0.309)
	Hippocampus	−0.005 (−0.010; 0.001)	−0.005 (−0.010; 0.000)
	Amygdala	−0.002 (−0.005; 0.001)	−0.002 (−0.005; 0.001)
	Thalamus	0.001 (−0.007; 0.010)	0.002 (−0.007; 0.010)

*Note*: Mean difference represents the difference in change in mL brain volume per year compared to the reference group; no use of any type of antidepressant is used as reference throughout. Model 1 is adjusted for age, sex, and education. Model 2 is adjusted for age, sex, education, intracranial volume, smoking status, alcohol use, body mass index, estimated glomerular filtration rate, Center for Epidemiologic Studies Depression Scale score, benzodiazepine use, antipsychotic medication use, and prevalence of diabetes, hypertension, stroke, parkinsonism, atrial fibrillation, congestive heart failure, coronary heart disease, cancer, and chronic obstructive pulmonary disease.

Abbreviation: CI, confidence interval.

### 3.4 Instrumental variable analyses

A higher CYP2D6 risk score, reflecting slower drug metabolism rate, was not associated with an increase in dementia risk among amitriptyline users (HR [95% CI]: 0.90 [0.80–1.01]) or paroxetine users (HR: 0.94 [0.81–1.09]). Adjusting for dose and co‐medication did not change these results (HR in amitriptyline users: 0.90 [0.76–1.02], and in paroxetine users: 0.83 [0.67–1.02]).

## 4 DISCUSSION

In this study, antidepressant use was not related to an increased dementia risk, cognitive decline, or brain atrophy in older individuals without indication of cognitive impairment.

The absence of a consistent association with dementia risk, cognitive decline, and brain atrophy is in concordance with earlier findings.^15^, ^16^, ^17^ In contrast, other studies did link antidepressant use to increased dementia risk.^4^, ^8^, ^9^, ^10^, ^11^, ^12^, ^13^, ^14^ Differences in environmental and genetic factors between study populations might have influenced results. In addition, because depression is a risk factor for dementia, antidepressants prescribed for depression might show an association with increased dementia risk even in the absence of a causal relationship (i.e., confounding‐by‐indication). Furthermore, depressive symptoms often occur in the prodromal phase of dementia as psychological reaction to cognitive decline or due to shared neurobiological mechanisms between depression and dementia, leading to increased antidepressant prescription in response to prodromal neurodegenerative disease (i.e., reversed causation),^42^, ^43^ supported by the somewhat higher estimates in current drug users (compared to past users) in the current study. We attempted to minimize reversed causation by stratifying by (history and severity of) depression and excluding participants with cognitive impairment at baseline.

Among older individuals, prevalence of clinical depression is high, with 7.5% of women and 5.5% of men affected worldwide, rising to one third in individuals with mild cognitive impairment.^44^ Older adults are more vulnerable to adverse effects,^45^ and equally effective non‐pharmacological treatments, such as cognitive behavioral therapy, often are available.^46^, ^47^ Yet, most of the patients are treated with medication, which in 30%–50% of cases is continued without an indication to continue.^48^, ^49^ Risk estimates for dementia in our study were somewhat higher for TCA users, but a causal association was not supported by a dose–response relationship, and no associations were observed with subclinical outcomes or in instrumental variable analysis. In the cognition analyses, the CIs do not rule out the possibility of a potential protective effect of antidepressant use on cognitive decline; however, the absence of any protective effect on dementia risk, brain MRI, and in instrumental variable analyses, and the absence of dose–response and duration–response relationships between antidepressant use and cognitive decline do not support a protective effect of antidepressant use on cognition. Although prescription of antidepressant medication in older individuals, in particular those with some cognitive impairment, may have acute symptomatic anticholinergic effects that warrant consideration in clinical practice, our results show that long‐term antidepressant use does not have lasting effects on cognition or brain health in older adults without indication of cognitive impairment.

Our study was strengthened by its linkage with pharmacy records, which provided detailed information on use up to 15 years before baseline, the availability of genetic data as proxy for metabolic rate, and the 10–15 years follow‐up on cognitive decline and brain atrophy. A post hoc power analysis showed that with our sample of 5511 participants, we had 80% power to detect a difference of 1.40 in the HR for dementia with any versus no use of antidepressants. There are limitations to consider also. First, the exclusion of participants with MMSE <26 at baseline prevented reversed causation (i.e., the use of antidepressants due to depression in the prodromal phase of dementia) but may have introduced selection bias by disregarding the effects of antidepressant use prior to baseline and excluding participants with lower education. Second, although attrition for dementia follow‐up was only 3.2%, a higher share of participants did not undergo repeated cognitive assessment or brain MRI (27% and 35%, respectively). Because individuals with severe mental health disorders and cognitive impairment might be more likely to drop out, this may have attenuated the association with cognitive decline and brain volume. Third, we were unable to include the presence of gene deletions or duplications in the CYP2D6 genotype in the genetic risk score, which may introduce a degree of misclassification. Fourth, results were obtained in a predominantly White population, and results may, therefore, be less generalizable to non‐White populations.

In conclusion, in this population‐based study, antidepressant use was not associated with long‐term adverse effects on dementia risk, cognitive decline, or brain atrophy in older individuals without clear signs of cognitive impairment.

## CONFLICT OF INTEREST STATEMENT

All authors have completed the ICMJE uniform disclosure form at http://www.icmje.org/disclosure‐of‐interest/ and declare: no support from any organization for the submitted work; no financial relationships with any organizations that might have an interest in the submitted work in the previous 3 years; and no other relationships or activities that could appear to have influenced the submitted work. Author disclosures are available in the supporting information.

## DETAILS OF ETHICAL APPROVAL

The Rotterdam Study has been approved by the Medical Ethics Committee of the Erasmus MC (registration number MEC 02.1015) and by the Dutch Ministry of Health, Welfare, and Sport (Population Screening Act WBO, license number 1071272‐159521‐PG). The Rotterdam Study has been entered into the Netherlands National Trial Register (NTR; www.trialregister.nl) and into the WHO International Clinical Trials Registry Platform (ICTRP; www.who.int/ictrp/network/primary/en/) under shared catalogue number NTR6831.

## CONSENT STATEMENT

All participants provided written informed consent to participate in the study after the procedures had been fully explained, and to have their information obtained from treating physicians.

## Supporting information

Supporting information

Supporting information

Supporting information

Supporting information

Supporting information

Supporting information
