# Supplementary material for: Antidepressant use in relation to dementia risk, cognitive decline, and brain atrophy
Source: Alzheimers Dement. 2024 Apr 1;20(5):3378–87. doi: 10.1002/alz.13807 (PMC11095425; doi:10.1002/alz.13807)
Supplement: Supplementary file 1 — Supporting information [file ALZ-20-3378-s006.pdf]

Figure S1. Overview of the study design and inclusion criteria.

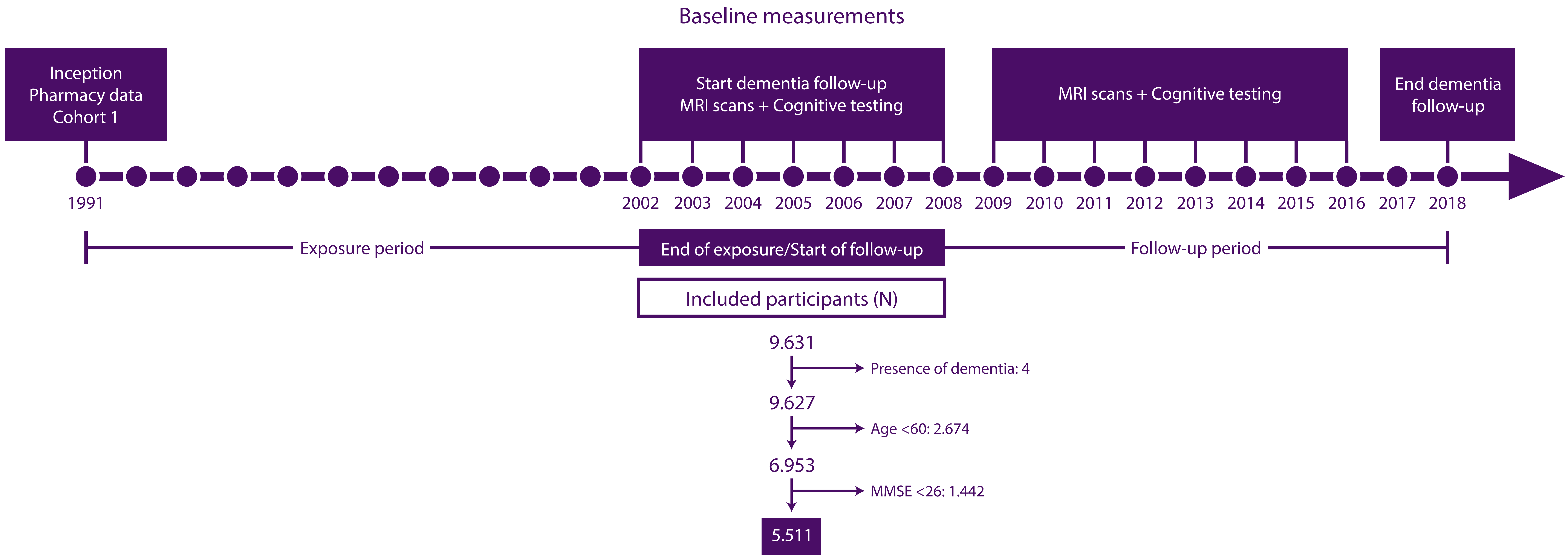

Pharmacy registry data were complete for all participants. Follow-up for dementia was complete for 96.8% of the potential person years.
