# Supplementary material for: Antidepressant use in relation to dementia risk, cognitive decline, and brain atrophy
Source: Alzheimers Dement. 2024 Apr 1;20(5):3378–87. doi: 10.1002/alz.13807 (PMC11095425; doi:10.1002/alz.13807)
Supplement: Supplementary file 2 — Supporting information [file ALZ-20-3378-s002.pdf]

Figure S2. Antidepressant medication use and change in standardized test scores over time.

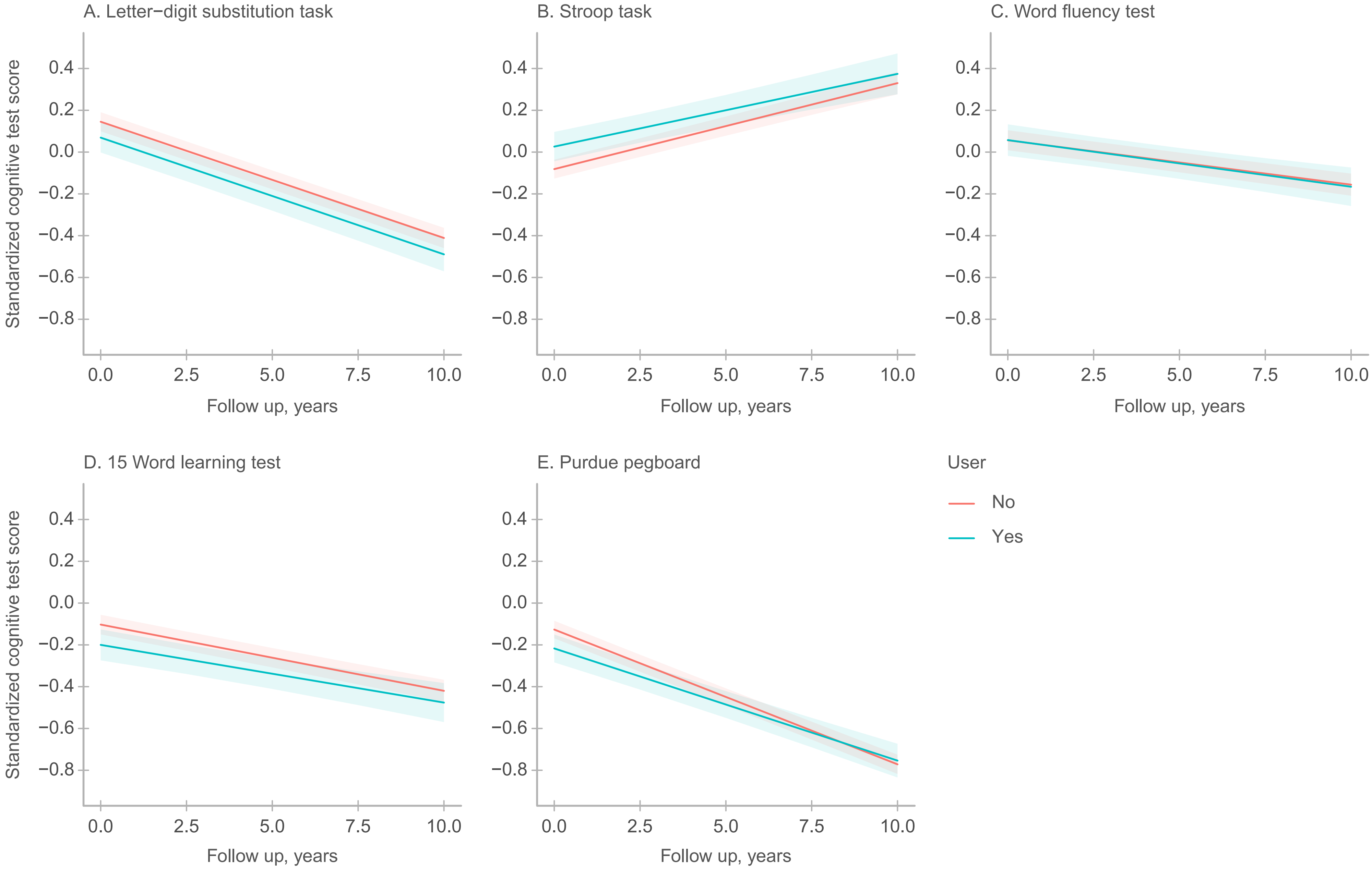

Trajectories show change in standardized test performance per year for ever users and never users. Performance on the letter-digit substitution task was defined as number of correct digits in 1 minute, on the Stroop test as error adjusted seconds at trial 3, on the word fluency task as number of animals in 1 minute, on the 15 word learning task as number of words recalled after delay, and on the purdue pegboard task as number of correct pins of left and right hand combined
