# Supplementary material for: Antidepressant use in relation to dementia risk, cognitive decline, and brain atrophy
Source: Alzheimers Dement. 2024 Apr 1;20(5):3378–87. doi: 10.1002/alz.13807 (PMC11095425; doi:10.1002/alz.13807)
Supplement: Supplementary file 3 — Supporting information [file ALZ-20-3378-s001.docx]

| **Table S1. Antidepressant use and risk of dementia.** | | | | | |
| --- | --- | --- | --- | --- | --- |
|  |  |  | **Crude model**  **HR (95% CI)** | **Model 1**  **HR (95% CI)** | **Model 2**  **HR (95% CI)** |
| **Antidepressants (any)** | Ever use |  | 1.25 (1.03 ; 1.52) | 1.24 (1.02 ; 1.51) | 1.14 (0.92 ; 1.41) |
|  | Duration of use | 1 – 90 days | 1.28 (0.95 ; 1.73) | 1.20 (0.89 ; 1.63) | 1.13 (0.82 ; 1.54) |
|  |  | > 90 days | 1.24 (0.98 ; 1.56) | 1.26 (1.00 ; 1.59) | 1.14 (0.89 ; 1.47) |
|  | Cumulative DDD | < median | 1.27 (0.98 ; 1.65) | 1.17 (0.90 ; 1.52) | 1.09 (0.83 ; 1.44) |
|  |  | > median | 1.24 (0.95 ; 1.61) | 1.32 (1.01 ; 1.72) | 1.19 (0.90 ; 1.57) |
|  |  |  |  |  |  |
| **Tricyclic antidepressants** | Ever use |  | 1.53 (1.15 ; 2.03) | 1.44 (1.08 ; 1.91) | 1.36 (1.01 ; 1.83) |
|  | Duration of use | 1 – 90 days | 1.59 (1.10 ; 2.30) | 1.43 (0.99 ; 2.07) | 1.42 (0.98 ; 2.08) |
|  |  | > 90 days | 1.46 (0.96 ; 2.22) | 1.45 (0.95 ; 2.20) | 1.29 (0.84 ; 1.98) |
|  | Cumulative DDD | < median | 1.63 (1.15 ; 2.30) | 1.47 (1.03 ; 2.08) | 1.43 (1.00 ; 2.04) |
|  |  | > median | 1.38 (0.88 ; 2.19) | 1.46 (0.92 ; 2.31) | 1.26 (0.78 ; 2.02) |
|  |  |  |  |  |  |
| **Serotonin reuptake inhibitors** | Ever use |  | 1.18 (0.87 ; 1.60) | 1.19 (0.88 ; 1.62) | 1.12 (0.81 ; 1.54) |
|  | Duration of use | 1 – 90 days | 1.05 (0.62 ; 1.79) | 1.05 (0.61 ; 1.78) | 0.92 (0.54 ; 1.59) |
|  |  | > 90 days | 1.25 (0.87 ; 1.79) | 1.24 (0.86 ; 1.78) | 1.23 (0.84 ; 1.79) |
|  | Cumulative DDD | < median | 1.18 (0.79 ; 1.75) | 1.17 (0.79 ; 1.74) | 1.08 (0.72 ; 1.62) |
|  |  | > median | 1.19 (0.75 ; 1.88) | 1.18 (0.74 ; 1.86) | 1.17 (0.73 ; 1.88) |
|  |  |  |  |  |  |
| **Combination of any type** | Ever use |  | 1.13 (0.78 ; 1.66) | 1.17 (0.80 ; 1.72) | 0.91 (0.60 ; 1.38) |
| Model 1 is adjusted for age, sex and education. Model 2 is adjusted for age, sex, education, smoking status, alcohol use, body mass index, estimated glomerular filtration rate, Center for Epidemiologic Studies Depression scale score, benzodiazepine use, antipsychotic medication use, and prevalence of diabetes, hypertension, stroke, parkinsonism, atrial fibrillation, congestive heart failure, coronary heart disease, cancer and chronic obstructive pulmonary disease. HR = hazard ratio. CI = confidence interval. DDD = defined daily dose. *No use of any type of antidepressant is used as reference throughout. | | | | | |
