# Supplementary material for: Antidepressant use in relation to dementia risk, cognitive decline, and brain atrophy
Source: Alzheimers Dement. 2024 Apr 1;20(5):3378–87. doi: 10.1002/alz.13807 (PMC11095425; doi:10.1002/alz.13807)
Supplement: Supplementary file 4 — Supporting information [file ALZ-20-3378-s005.docx]

| **Table S2. Antidepressant use and change in global cognition (g-factor) over time.** | | | | | |
| --- | --- | --- | --- | --- | --- |
|  |  |  | **Crude model**  **Mean difference**  **(95% CI)** | **Model 1**  **Mean difference**  **(95% CI)** | **Model 2**  **Mean difference**  **(95% CI)** |
| **Antidepressants (any)** | Ever use |  | 0.005 (-0.002 ; 0.012) | 0.003(-0.003 ; 0.010) | 0.003 (-0.003 ; 0.010) |
|  | Duration of use | 1 – 90 days | 0.006 (-0.005 ; 0.017) | 0.007 (-0.004 ; 0.017) | 0.003 (-0.006 ; 0.012) |
|  |  | > 90 days | 0.005 (-0.004 ; 0.013) | 0.001 (-0.007 ; 0.010) | 0.003 (-0.006; 0.013) |
|  | Cumulative DDD | < median | 0.003 (-0.007 ; 0.012) | 0.012 (-0.175 ; 0.200) | 0.003 (-0.006 ; 0.012) |
|  |  | > median | 0.008 (-0.002 ; 0.017) | 0.000 (-0.188 ; 0.189) | 0.003 (-0.006 ; 0.013) |
|  |  |  |  |  |  |
| **Tricyclic antidepressants** | Ever use |  | 0.009 (-0.003 ; 0.020) | 0.010 ( -0.001 ; 0.021) | 0.010 (-0.001 ; 0.021) |
|  | Duration of use | 1 – 90 days | 0.003 (-0.012 ; 0.019) | 0.006 (-0.009 ; 0.021) | 0.006 (-0.009 ; 0.021) |
|  |  | > 90 days | 0.015 (-0.002 ; 0.031) | 0.014 (-0.001 ; 0.030) | 0.014 (-0.002 ; 0.030) |
|  | Cumulative DDD | < median | 0.008 (-0.007 ; 0.023) | 0.011 (-0.003 ; 0.025) | 0.011 (-0.003 ; 0.025) |
|  |  | > median | 0.010 (-0.008 ; 0.027) | 0.009 (-0.008 ; 0.026) | 0.008 (-0.009 ; 0.025) |
|  |  |  |  |  |  |
| **Serotonin reuptake inhibitors** | Ever use |  | 0.008 (-0.003 ; 0.019) | 0.006 (-0.004 ; 0.017) | 0.006 (-0.004 ; 0.017) |
|  | Duration of use | 1 – 90 days | 0.014 (-0.004 ; 0.032) | 0.015 (-0.002 ; 0.032) | 0.015 (-0.002 ; 0.032) |
|  |  | > 90 days | 0.005 (-0.008 ; 0.018) | 0.002 (-0.011 ; 0.014) | 0.002 (-0.011 ; 0.014) |
|  | Cumulative DDD | < median | 0.008 (-0.006 ; 0.022) | 0.007 (-0.006 ; 0.021) | 0.007 (-0.006 ; 0.020) |
|  |  | > median | 0.009 (-0.007 ; 0.026) | 0.005 (-0.011 ; 0.021) | 0.005 (-0.011 ; 0.021) |
|  |  |  |  |  |  |
| **Combination of any type** | Ever use |  | -0.007 (-0.021 ; 0.007) | -0.012 (-0.025 ; 0.001) | -0.012 (-0.025 ; 0.001) |
|  | Duration of use | 1 – 90 days | -0.019 (-0.066 ; 0.028) | -0.024 (-0.069 ; 0.021) | -0.024 (-0.069 ; 0.020) |
|  |  | > 90 days | -0.006 (-0.020 ; 0.008) | -0.011 (-0.024 ; 0.003) | -0.011 (-0.024 ; 0.003) |
|  | Cumulative DDD | < median | -0.037 (-0.065 ; -0.009) | -0.039 (-0.066 ; -0.012) | -0.039 (-0.065 ; -0.012) |
|  |  | > median | 0.002 (-0.014 ; 0.017) | -0.004 (-0.019 ; 0.011) | -0.004 (-0.019 ; 0.011) |
| Mean difference represents the difference in change in global cognition per year compared to the reference group, no use of any type of antidepressant is used as reference throughout. Global cognition is defined as the standardized compound score (g-factor) using the first factor of a principal component analysis, including the verbal fluency test, the Stroop test, the 15 word learning test and the purdue pegboard test. Model 1 is adjusted for age, sex and education. Model 2 is adjusted for age, sex, education, smoking status, alcohol use, body mass index, estimated glomerular filtration rate, Center for Epidemiologic Studies Depression scale score, benzodiazepine use, antipsychotic medication use, and prevalence of diabetes, hypertension, stroke, parkinsonism, atrial fibrillation, congestive heart failure, coronary heart disease, cancer and chronic obstructive pulmonary disease. CI = confidence interval. DDD = defined daily dose. | | | | | |
