# Supplementary material for: Antidepressant use in relation to dementia risk, cognitive decline, and brain atrophy
Source: Alzheimers Dement. 2024 Apr 1;20(5):3378–87. doi: 10.1002/alz.13807 (PMC11095425; doi:10.1002/alz.13807)
Supplement: Supplementary file 5 — Supporting information [file ALZ-20-3378-s003.docx]

| **Table S3. Antidepressant use (any type) and change in mL brain volume over time.** | | | | |
| --- | --- | --- | --- | --- |
| **Brain region** | **Medication use** | **Crude model**  **Mean difference**  **(95% CI)** | **Model 1**  **Mean difference**  **(95% CI)** | **Model 2**  **Mean difference**  **(95% CI)** |
| Total brain | Antidepressants (ever vs none) | 0.027 (-0.213 ; 0.267) | -0.042 (-0.327 ; 0.244) | -0.000 (-0.276 ; 0.276) |
| Grey matter | Antidepressants (ever vs none) | -0.061 (-0.189 ; 0.067) | 0.084 (-0.316 ; 0.483) | 0.066 (-0.312 ; 0.444) |
| White matter | Antidepressants (ever vs none) | 0.082 (-0.092 ; 0.257) | -0.099 (-0.483 ; 0.285) | -0.051 (-0.422 ; 0.321) |
| Hippocampus | Antidepressants (ever vs none) | -0.004 (-0.008 ; 0.001) | -0.004 (-0.008 ; 0.001) | -0.004 (-0.008 ; 0.000) |
| Amygdala | Antidepressants (ever vs none) | -0.001 (-0.004 ; 0.002) | -0.001 (-0.004 ; 0.002) | -0.001 (-0.004 ; 0.002) |
| Thalamus | Antidepressants (ever vs none) | 0.001 (-0.006 ; 0.008) | 0.001 (-0.005 ; 0.008) | 0.002 (-0.005 ; 0.009) |
| Mean difference represents the difference in change in mL brain volume per year compared to the reference group, no use of any type of antidepressant is used as reference throughout. Model 1 is adjusted for age, sex and education. Model 2 is adjusted for age, sex, education, intracranial volume, smoking status, alcohol use, body mass index, estimated glomerular filtration rate, Center for Epidemiologic Studies Depression scale score, benzodiazepine use, antipsychotic medication use, and prevalence of diabetes, hypertension, stroke, parkinsonism, atrial fibrillation, congestive heart failure, coronary heart disease, cancer and chronic obstructive pulmonary disease . CI = confidence interval. | | | | |
